# A Non-Invasive Method to Assess Cerebral Perfusion Pressure in Geriatric Patients with Suspected Cerebrovascular Disease

**DOI:** 10.1371/journal.pone.0120146

**Published:** 2015-03-19

**Authors:** Bo Liu, Qi Li, Kewei Li, Nan Deng, Peng He, Chunchang Qin, Deyu Yang, Zhiwei Li, Peng Xie

**Affiliations:** 1 Department of Neurology, Yong Chuan Hospital, Chongqing Medical University, Chongqing, People’s Republic of China; 2 Department of Neurology, The First Affiliated Hospital of Chongqing Medical University, Chongqing, People’s Republic of China; 3 Institute of Neuroscience, Chongqing Medical University, Chongqing, People’s Republic of China; 4 Chongqing Key Laboratory of Neurobiology, Chongqing Medical University, Chongqing, People’s Republic of China; 5 Department of Gastroenterology, The First Affiliated Hospital of Chongqing Medical University, Chongqing, People’s Republic of China; 6 Department of Cardiology, The First Affiliated Hospital of Chongqing Medical University, Chongqing, People’s Republic of China; University at Buffalo, UNITED STATES

## Abstract

**Background:**

Cerebral perfusion pressure (CPP) can adversely impact cerebrovascular hemodynamics but cannot be practically measured in most clinical settings. Here, we aimed to establish a representative mathematical model for CPP in geriatric patients with suspected cerebrovascular disease.

**Methods:**

A total of 100 patients (54 males and 46 females between 60–80 years of age) with suspected cerebrovascular disease and no obvious cerebrovascular stenosis were selected for invasive CPP monitoring via catheterization of the middle segment of the common carotid arteries and openings of the vertebral arteries bilaterally. Curves were function-fitted using MATLAB 7.0, and data was statistically processed by SPSS 20.0.

**Results:**

MATLAB 7.0 constructed eighth-order Fourier functions that fit all recorded CPP curves. Since the coefficients of the 100 functions were significantly different, all functions were standardized to derive one representative function. By manipulating the heart rate and maximum/minimum CPP of the representative function, estimated CPP curves can be constructed for patients with differing heart rates, intracranial pressures (ICPs) and blood pressures.

**Conclusions:**

CPP can be well-modeled through an eighth-order Fourier function that can be constructed from a patient’s brachial artery blood pressure (BABP), ICP and heart rate. This function is representative of geriatric patients with cerebrovascular disease and can be used in the future study of cerebral hemodynamics.

## Introduction

The incidence of cerebrovascular disease has been steadily increasing year-by-year and has become a serious public health problem[1,2]. Previous studies have shown that cerebrovascular disease is closely related to hypertension, because high blood pressure significantly increases cerebral perfusion pressure (CPP), resulting in cerebrovascular overload and pathological vascular changes such as aneurysm, vascular stenosis, and occlusion. These phenomena can eventually result in cerebral infarction and cerebral hemorrhage[3,4]. Therefore, CPP can adversely impact cerebrovascular hemodynamics and should be an important focus of investigation in patients with cerebrovascular disease.

However, CPP cannot be practically measured in most clinical settings, making CPP an inconvenient measure for the study of cerebrovascular hemodynamics[5,6]. In 1966, Attinger et al proposed that Fourier series could effectively be used to simulate arterial blood pressure via a sixth-order Fourier function[7]. However, the fitting method proposed by Attinger and colleagues could only be performed by artificial calculation, making the functional coefficients difficult to calculate. To address this issue, our study aims to establish a representative mathematical model to simulate the CPP in a cohort of geriatric patients with suspected cerebrovascular disease.

## Methods

All subjects gave their written informed consent after a detailed introduction to the study. The study was approved by the Ethics Committee of Chongqing Medical University (Approval number: 20110118) and was performed according to the Helsinki Declaration.We have included patients with suspected cerebrovascular disease who underwent CPP monitoring and digital subtraction angiography (DSA) in our institution between January 2011 and August 2013. The inclusion criteria was as follows: 1) patients had signs and symptoms suggestive of cerebrovascular disease. 2) patients had to complete DSA examination for visualization of cerebral vasculatures. 3) patients had to complete CPP monitoring for inclusion. 4) in the included patients, the CPP_max_ /CPP_min_ range was 80–180/50–110 mm Hg, the intracranial pressure (ICP) range was 5.88–13.24 mm Hg and the heart rate range was 60–100 beats/min. Patients were excluded from the study if they had severe steno-occlusive disease of the carotid and vertebrobasilar artery. Patients with recent onset moderate to severe stroke which is defined as NIHSS score>4 points were excluded from the study. Patients with Arrhythmia were excluded from the study.

After completing the DSA, a total of four CPP monitoring curves were bilaterally recorded for each patient: two from the (left and right) common carotid arteries and two from the openings of the (left and right) vertebral arteries were obtained. Briefly, angiographic catheters were inserted into the middle segments of the common carotid arteries bilaterally and openings of the vertebral arteries (initial segment of bilateral subclavian artery) bilaterally, then the carotid and vertebral artery pressure (CVAP) was monitored. The intracranial pressure(ICP) was monitored by using non-invasive methods. First, the pulsatility index (PI) of the middle cerebral artery was detected by using Transcranial Doppler (TCD) in each patient. The mean value of the PI was calculated by using the two PI on both sides. The ICP was calculated by using the equation“ICP = 10.927* PI—1.284” [8,9]. CPP (CPP = CVAP-ICP) was monitored and recorded by a multichannel physiologic recorder (Sichuan Jinjiang Electronic Science and Technology Co.). CPP was determined by the consensus reading of three readers. CPP waveform was observably stable for one minute.

All curves were analyzed by Jinjiang multi-channel physiological analysis software (Lead System). The most stable segments from each recording were judged by the consensus reading of two reviewers and saved as PNG images. The color PNG images were converted into black-and-white JPG images by using Adobe Photoshop 7.0.1. Using the JPG images, points on the CPP curves were automatically captured by GetData Graph Digitizer 2.25 after setting the accurate coordinates. The software captures 3 waves at one time and each wave was consisted of 40 points. For each CPP curve, the coordinate figures at each point were gained and saved as an Excel data series. The Excel data was then function-fitted using MATLAB 7.0's Curve Fitting Toolbox with the use of the least square method[10,11].

CPP wave is a irregular periodic wave. According to the Parseval’s theorem, periodic signal can be modeled as the superposition of all the harmonics by using the triangular form of the Fourier series ($f(t)=a_{0}+\sum_{n=1}^{\infty }(a_{n}\cos nwt+b_{n}\sin nwt)$,n = 1,2,3…) [12]. The eighth-order Fourier function ($f(t)=a_{0}+\sum_{n=1}^{8}(a_{n}\cos nwt+b_{n}\sin nwt)$ was chosen for each patient CPP curve fitting function. The bandwidth of the curve was set at ± 2mmHg (ordinate). All raw data points were located within the bandwidth. An eighth-order Fourier function is merely the sum of eight constituent sine functions and eight constituent cosine functions with *a*_*0*_ as the average[12].

By setting the time of the nadir at 0 s, four Fourier functions (i.e., left and right carotid arteries, *f*_*LCCA*_(*t*) and *f*_*RCCA*_(*t*), left and right vertebral arteries, *f*_*LVA*_ *(t)* and *f*_*RVA*_ *(t)*) were obtained for each patient. The mean, maximum, and minimum of each cycle for each function were calculated by MATLAB 7.0.

$$\bar{CPP}=a_{0}$$

$$CPP_{\min}=a_{\text{0}}\text{+}a_{\text{1}}\text{+}a_{\text{2}}\text{+}a_{\text{3}}\text{+}a_{\text{4}}\text{+}a_{\text{5}}\text{+}a_{\text{6}}\text{+}a_{\text{7}}\text{+}a_{\text{8}}$$

$$CPP_{\max}\text{=}a_{0}+\sum_{n=1}^{8}(a_{n}\cos nwt_{\max}+b_{n}\sin nwt_{\max})$$

$$T_{\text{cycle}}=\frac{2\pi }{W}$$

### Statistical Analysis

All statistical analysis was performed by using SPSS 20.0. The one-way analysis of variance was employed to examine the group differences. A p value <0.05 was considered statistically significant.

**Fig 1:**
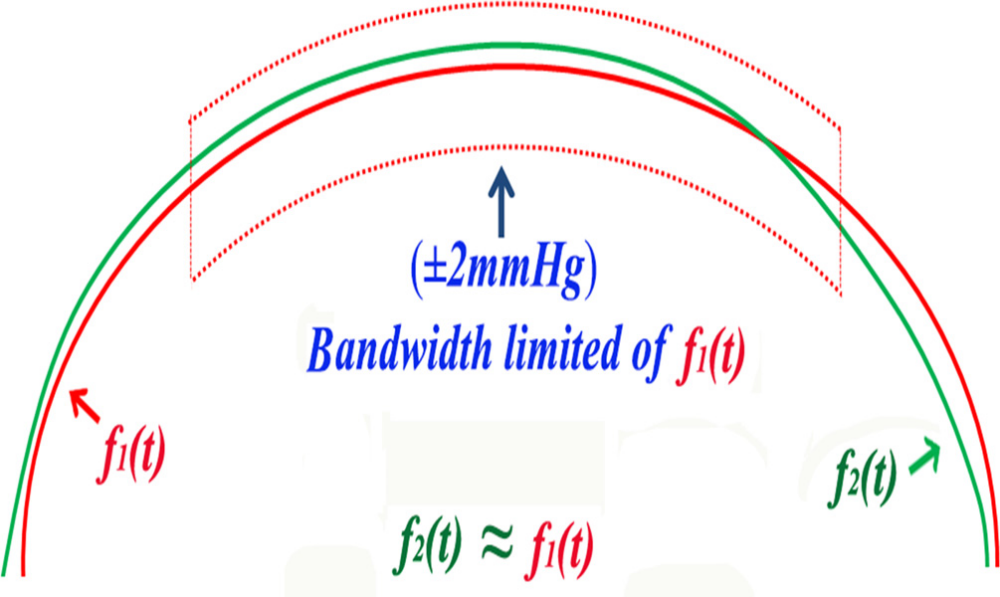
A function travels within bandwidth of another function.

Definite integral of the absolute value of the difference function in [0,1] was used to compare the similarity function. The single sample Kolmogorov-Smirnov test was employed to test $\int_{{}_{0}}^{1}\left|f_{LCCA}(t)-f_{RCCA}(t)\right|dx$,$\int_{{}_{0}}^{1}\left|f_{LCCA}(t)-f_{LVA}(t)\right|dx$, $\int_{{}_{0}}^{1}\left|f_{LCCA}(t)-f_{RVA}(t)\right|dx$, $\int_{{}_{0}}^{1}\left|f_{RCCA}(t)-f_{LVA}(t)\right|dx$,$\int_{{}_{0}}^{1}\left|f_{RCCA}(t)-f_{RVA}(t)\right|dx$, $\int_{{}_{0}}^{1}\left|f_{LVA}(t)-f_{RVA}(t)\right|dx$ in all patients. The 95% confidence interval <2 is considered the same curve (Fig. 1).

## Results

**Fig 2:**
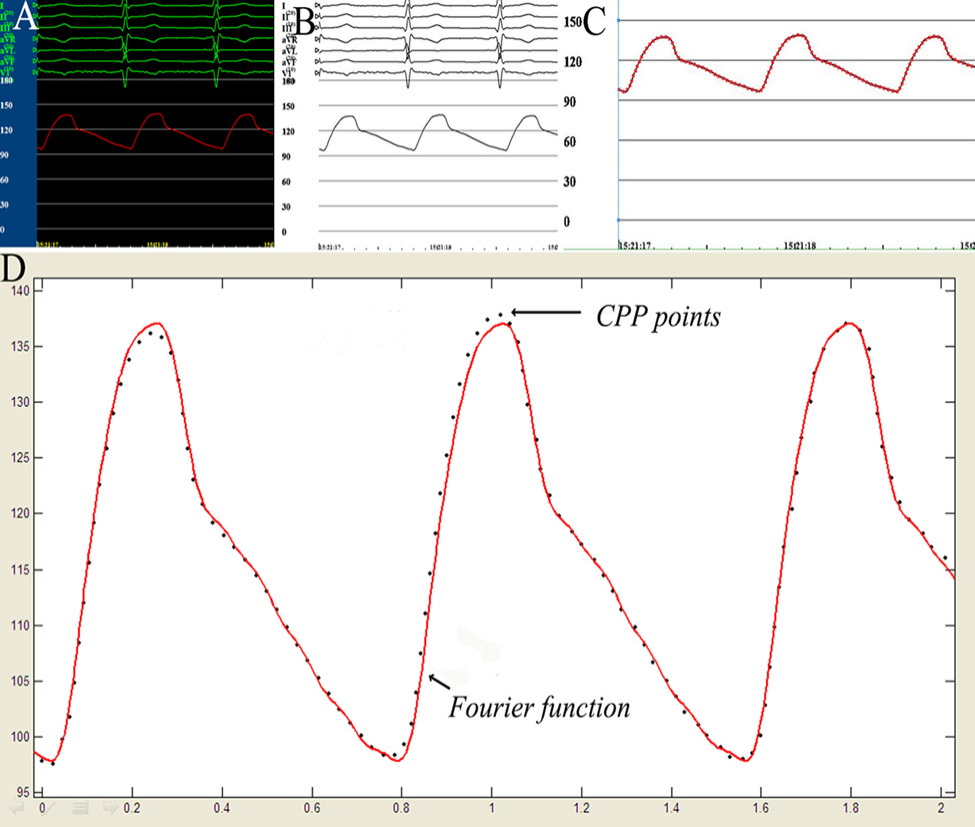
Application of MATLAB 7.0,Photoshop 7.0.1 and GetData Graph Digitizer 2.25 to construct a Fourier function fitting a CPP curve.

A total of 100 patients were included into our study. The demographic data was listed in Table 1. The majority of the 100 patients suffered from hypertension. Likely on account of psychological stress during the invasive procedure, the CPP was usually higher than the normal range with a maximum/minimum range of 100–180/60–110 mm Hg. Although some patients did use antihypertensive agents during the operation, it was difficult to control the CPP to under 140/90 mm Hg. Relatively stable CPP curve was generated in each patient who underwent DSA examination (Fig. 2A), CPP image was converted in the Adobe Photoshop 7.0.1 without data loss (Fig. 2B), GetData Graph Digitizer 2.25 captured CPP data points on each curve (Fig. 2C), CPP curve representative data points were well function-fitted using MATLAB 7.0 (Fig. 2D).

**Table 1:** Demographics of the patients studied.

Item	value
**Mean age_SD (range)**	69_5(60–80)
**Men, n (%)**	54(54)
**Women, n (%)**	46(46)
**Hypertension, n (%)**	95(95)
**Diabetes mellitus, n (%)**	13(13)
**Hypercholesterolemia, n(%)**	26(26)
**Current smokers, n (%)**	21(21)

**Fig 3:**
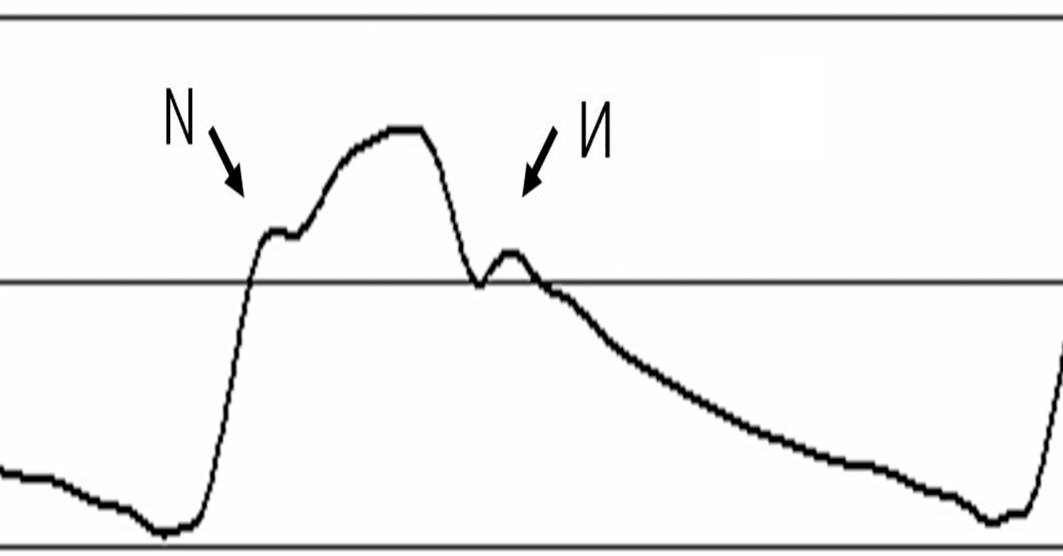
A CPP curve displays the characteristic “N” and “И”.

Based on all 100 CPP curves, the maximum, minimum, and rhythm of the CPP displayed significant differences across patients; however, their overall patterns were similar. The curve starts at its nadir, then rapidly rises, and reaches its climax at a slowing rate of speed. Some patients' CPP curves show a large angle between fast-rising and slow-rising phases, even forming an “N”-shaped barb in select cases (Fig. 3). After climaxing, the CPP curve rapidly declines and then slows its rate of decline until reaching its nadir. During the CPP curve's descending phase, some patients' CPP curves form a dicrotic notch, similar in shape to the Cyrillic letter “И” (Fig. 3). When a patient’s CPP curve shows an “N”-shaped rise or “И”-shaped decline, the fitted function was still largely consistent with the original curve.

MATLAB 7.0 was able to construct eighth-order Fourier functions to fit all recorded CPP curves. In each patient, there were no significant differences in the appearances across the four CPP curves (i.e., left and right carotid arteries, left and right vertebral arteries) (*p*>0.05; Tables 2 and 3). Therefore, *f*_*LCCA*_(*t*) was used to represent each patient's CPP, yielding 100 Fourier functions (exactly one function per patient). Due to differing heart rates,ICPs and blood pressures as well as variations in heart and blood vessel structure across patients, each of these 100 *f*_*LCCA*_(*t*) had their own unique coefficients (i.e., *w*, *a*_*0*_ *-a*_*8*_, *b*_*1*_ *-b*_*8*_).

**Table 2:** One-way ANOVA of the $\bar{CPP}$,*CPP*_*min*_ and *CPP*_*max*_ $\left(\bar{x}\pm s,n=100\right)$.

Item	Mean±S				*P*-value
	RCCA	LCCA	RVA	LVA	
$\bar{CPP}$	109.02±9.60	110.64±10.68	110.76±11.77	111.14±12.03	>0.05
***CPP***_***min***_	88.02±9.18	89.59±8.86	89.25±9.18	88.23±9.87	>0.05
***CPP***_***max***_	136.35±19.09	135.97±18.50	137.55±19.54	137.61±18.96	>0.05

**Table 3:** The single sample Kolmogorov-Smirnov test of six definite integrals in 100 patients and 95% confidence interval $\left(\bar{x}\pm s,n=100\right)$.

Definite integral	*P*-value	Mean±S	Confidence interval(95%)	The upper boundary value
$\int_{{}_{0}}^{1}\left|f_{LCCA}(t)-f_{RCCA}(t)\right|dx$	>0.05	1.57±0.49	[1.47, 1.66]	<2
$\int_{{}_{0}}^{1}\left|f_{LCCA}(t)-f_{LVA}(t)\right|dx$	>0.05	1.56±0.52	[1.45, 1.66]	<2
$\int_{{}_{0}}^{1}\left|f_{LCCA}(t)-f_{LVA}(t)\right|dx$	>0.05	1.65±0.49	[1.56, 1.75]	<2
$\int_{{}_{0}}^{1}\left|f_{LCCA}(t)-f_{LVA}(t)\right|dx$	>0.05	1.52±0.54	[1.42, 1.62]	<2
$\int_{{}_{0}}^{1}\left|f_{RCCA}(t)-f_{RVA}(t)\right|dx$	>0.05	1.48±0.47	[1.38, 1.56]	<2
$\int_{{}_{0}}^{1}\left|f_{LCCA}(t)-f_{LVA}(t)\right|dx$	>0.05	1.70±0.45	[1.61, 1.79]	<2

**Fig 4:**
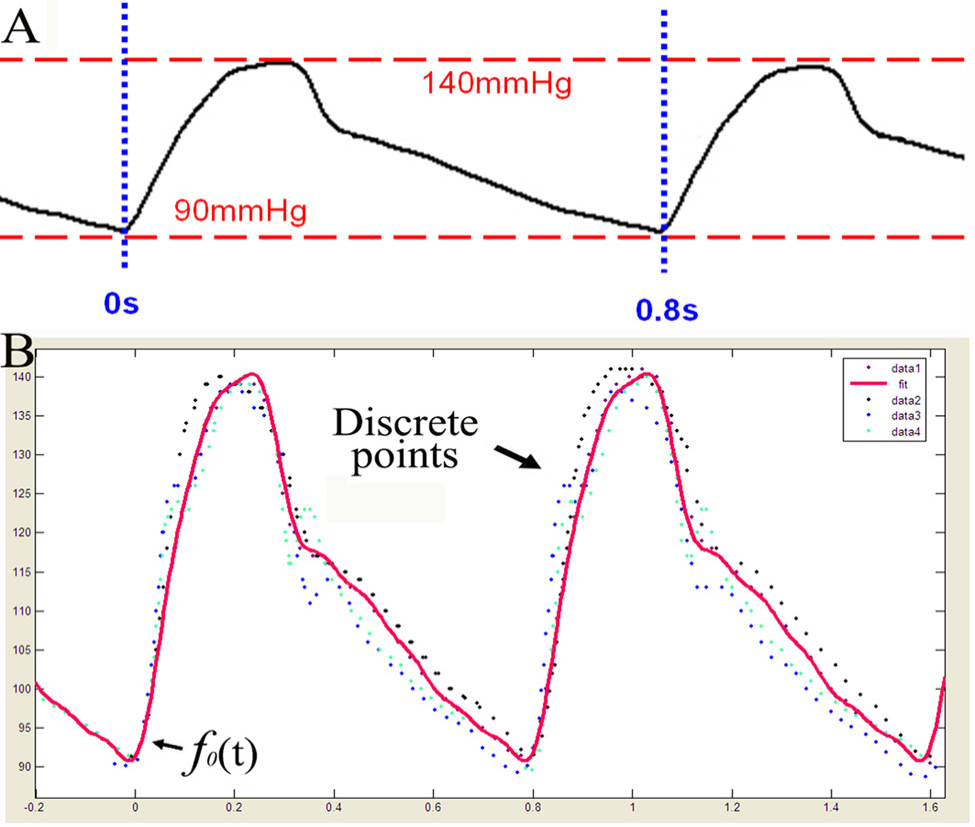
Standardization of CPP curves(A);Representative Fourier function *f*_*0*_ *(t)* (in red) matches with the overall trend of the 100 fitted functions after standardization (B).

Since the 18 coefficients (i.e., *w*, *a*_*0*_ *-a*_*8*_, *b*_*1*_ *-b*_*8*_) were significantly different across the 100 patients, it was necessary to standardize the 100 unique *f*_*LCCA*_ *(t)* to derive one representative Fourier function with 18 representative coefficients. In order to do so, all 100 CPP curves of LCCA were standardized through the following method: (1) the abscissa (time) was set to 0 s at the first nadir; (2) the abscissa (time) was set to 0.8 s at the second nadir; (3) the ordinate of the nadir was set to 90 mm Hg; and (4) the ordinate of the zenith was set to 140 mm Hg (Fig. 4A). Thus, this procedure standardized all cardiac cycles to 0.8 s and all *CPP*_*max*_ /*CPP*_*min*_ to 140/90 mm Hg. GetData Graph Digitizer 2.25 was then used to apply the newly standardized coordinates and capture the data points on all 100 newly standardized CPP curves. MATLAB 7.0 was then used for function fitting. After this standardization procedure, the 18 standardized coefficients (i.e., *w*, *a*_*0*_ *-a*_*8*_, *b*_*1*_ *-b*_*8*_) were obtained by calculating the mean of each coefficient of 100 new fitted functions (Table 4).

**Table 4:** The mean value of each coefficient of 100 CPP fitted functions after standardization.

Coefficient	Mean±S	Coefficient	Mean±S
*a*_*0*_	112.3±2.03	*w*	7.85±0.02
*a*_*1*_	-10.37±2.32	*b*_*1*_	17.27±3.51
*a*_*2*_	-8.731±1.01	*b*_*2*_	0.6382±0.75
*a*_*3*_	-0.9819±0.37	*b*_*3*_	-2.315±0.80
*a*_*4*_	-1.086±0.36	*b*_*4*_	0.3577±0.24
*a*_*5*_	-1.098±0.52	*b*_*5*_	-1.342±0.80
*a*_*6*_	0.3895±0.24	*b*_*6*_	-0.4227±0.13
*a*_*7*_	-0.4628±0.25	*b*_*7*_	0.02835±0.53
*a*_*8*_	0.04679±0.33	*b*_*8*_	-0.7078±0.84

Based on the standardized coefficients in Table 4, we derived the following representative eighth-order Fourier function: *f*_*0*_ *(t)* = 112.3 –[10.37cos(7.85*t*) –17.27sin(7.85*t*)]–[8.731cos(2*7.85*t*)– 0.6382sin(2*7.85*t*)]–[0.9819cos(3*7.85*t*)+2.315sin(3*7.85*t*)]–[1.086cos(4*7.85*t*)– 0.3577sin(4*7.85*t*)]–[1.098cos(5*7.85*t*)+1.342sin(5*7.85*t*)] + [0.3895cos(6*7.85*t*)– 0.4227sin(6*7.85*t*)]–[0.4628cos(7*7.85*t*)– 0.02835sin(7*7.85*t*)] + [0.04679cos(8*7.85*t*)– 0.7078sin(8*7.85*t*)]. As shown in Fig. 4B, the *f*_*0*_ *(t)* curve closely approximates the underlying data points (four representative data series of 100 new fitted functions) with a cycle of $T_{\text{0}}=\frac{2\pi }{7.85}\approx 0.8$s,${\bar{CPP}}_{\text{0}}\text{=112}\mathrm{.3}$ mm Hg, *CPP*_*min*_ = 90 mm Hg, *CPP*_*max*_ = 140 mm Hg.

**Fig 5:**
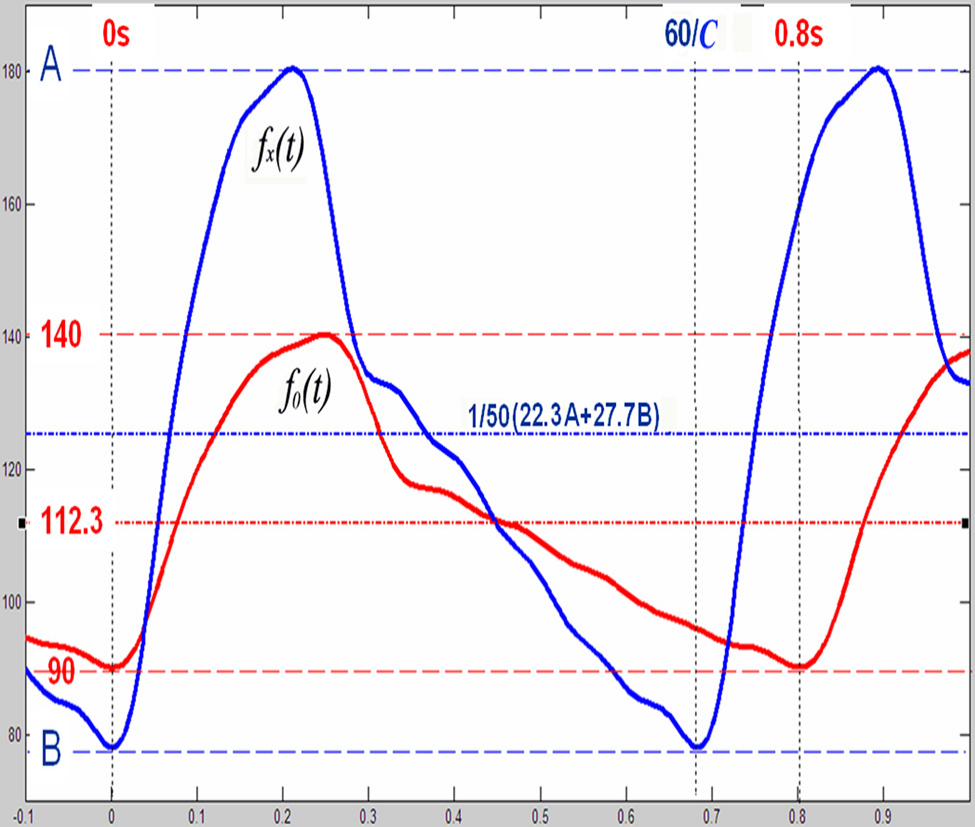
Graphical comparison of *f*_*0*_ *(t)* and *f*_*x*_ *(t)*.

*f*_*0*_ *(t)* displays a pendulum-like periodic motion around *a*_*0*_ (112.3 mm Hg) with its positive amplitude (27.7 mm Hg) slightly greater than its negative amplitude (23.3 mm Hg) (Fig. 5). By manipulating *a*_*0*_ and/or the amplitudes in the positive and negative directions, one can simulate a particular patient's *CPP* levels, and the *w* value can be changed to match that patient's heart rate. Thus, in an arbitrary patient *X*, we can define the *CPP*_*max*_ /*CPP*_*min*_ as *A/B* (mm Hg) and the heart rate as *C* (beats/min), yielding a cycle of $T_{\text{cycle}}=\frac{2\pi }{W}=\frac{60}{C},W=\frac{2\pi *C}{60}=1/30*C\pi$. According to the Fourier function, patient *X*'s CPP function *f*_*x*_ *(t)* should satisfy the following relationship:

$$f_{x}(t)=\frac{\text{22}.3A+27.7B}{50}+\frac{\text{A-B}}{50}*(f_{0}(t,C)-112.3)\;\text{(mm Hg)}\text{.}$$

Based on this formula, *f*_*x*_ *(t)* has an average value of (1/50)(22.3A + 27.7B) (mm Hg), an ordinate range from A to B, and a cycle of 60/C (s)(Fig. 5). As the construction of *f*_*x*_ *(t)* curve simply involves the lengthening and/or shortening of *f*_*0*_ *(t)* in the longitudinal and transverse directions, the *f*_*x*_ *(t)* function can be manipulated to fit the CPP curves for differing blood pressures,ICPs and heart rates. However, if a particular patient *X*’s blood pressure, ICP and/or the heart rate is too high or low, it will produce extreme variations in the CPP curve that may not be suitable for fitting using *f*_*x*_ *(t)*.

## Discussion

The French mathematician Fourier discovered that any periodic function can be expressed via an arithmetic sum of constituent sine and cosine functions. This concept is now commonly known as the Fourier series (French: *Série de Fourier*)[12]. In the field of medicine, Fourier transforms are primarily used in data acquisition by medical equipment and data processing of rhythmic physiological processes, such as cardiovascular hemodynamics[12,13]. The cerebrovascular trunk includes the bilateral common carotid arteries and bilateral vertebral arteries, all four of which originate from the aortic arch. Therefore, the CPP is directly affected by the aortic arch's blood pressure and is a cyclical function subject to Fourier transform. Unfortunately, the CPP cannot be practically measured in most mundane clinical situations. Although the CPP can be estimated by taking a non-invasive brachial artery blood pressure(BABP) or non-invasive applanation tonometry of the carotid artery pressure(ATCAP) and ICP, the result is rather unreliable. Therefore, the aim of this study was to: (1) measure the complex variations in CPP by invasive monitoring and (2) simulate these complex variations in the CPP curve via simple Fourier functions with a limited number of variables in order to (3) enable modeling of the CPP via a non-invasive check.

Normal ICP range is 5.88–13.24 mm Hg and is not easily affected by the heart rate. Under normal conditions it fluctuates between 2–4mmHg. In sharp contrast to the fluctuation of CVAP and CPP, the ICP curve could be regarded as a nearly straight line. In order to simplify calculations, we used ICP curve as a straight line. So CPP curve is like a downward shift of the CVAP curve. We have used non-invasvie methods for detection of ICP, which is expressed as a mean value. Although it might be less accurate than invasive measurement, the bias error is too small to affect our results because we have standardized all CPP_*max*_ /CPP_*min*_ to 140/90 mm Hg when calculating *f*_0_ *(x)*. Here, the authors invasively monitored the CPP(CPP = CVAP-ICP) of 100 patients; thus, the CPP curves accurately reflected the dynamic CPP changes in each patient.

CPP (and peripheral arterial blood pressures) are periodic, continuous, and smooth with no obvious breakpoints and buckling, generally conforming to a complex sinusoidal function. Moreover, the common carotid and cerebral arterial trees are unique anatomical structures; they are not simple cylindrical tubes attached to the aortic arch. Due to these underlying complexities in peripheral arterial blood flow and anatomical architecture, the CPP is an irregular sinusoidal curve that requires an eighth-order Fourier function for effective fitting[12,14].

This study found no significant differences among CPPs of the common carotid arteries and vertebral arteries in each patient on account of their shared source (the aortic arch) and close proximity. The *f*_*x*_ *(t)* function requires three variables: the patient's heart rate, the maximal CPP, and the minimal CPP. The heart rate can be easily assessed; however, this study used an invasive method to check the CPP that was only incidentally performed after DSA, which is not clinically feasible and thus not worth promoting as the basis of a practical diagnostic modality. Thus, the maximal and minimal CPP values can only be estimated through the patient’s non-invasive BABP and ICP(CPP_max_≈ BABP_max_-ICP, CPP_min_≈ BABP_min_-ICP), or non-invasive ATCAP and ICP(CPP_max_≈ ATCAP_max_-ICP, CPP_min_≈ ATCAP_min_-ICP). the accuracy of which depends on the measurement errors of BABP, ATCAP and/or ICP and the conditions of the patient's vasculature.

Patients involved in this research were between 60–80 years old, some of which suffered from hypertension. Therefore, in theory, the *f*_*x*_ *(t)* function obtained here is only applicable in this patient population. However, the majority of patients with cerebrovascular disease are in this age group, and most of them experience hypertension[15–17]. Therefore, the *f*_*x*_ *(t)* function derived here is of applicable value to cerebrovascular hemodynamic investigations in cerebrovascular disease patients. Further studies should apply the *f*_*x*_ *(t)* to investigating CPP changes in patients with cardiovascular disease and the role of CPP changes in the pathogenesis of cerebrovascular disease.

## Conclusions

CPP can be well-modeled through an eighth-order Fourier function using MATLAB 7.0. This function can be roughly estimated through non-invasively collecting the patient's BABP, ICP and heart rate. The Fourier function *f*_*x*_ *(t)* obtained here is widely representative of geriatric patients with cerebrovascular disease and can be used in the future study of cerebral hemodynamics. It also serves as a useful reference for further investigations on cerebrovascular disease.
